# Genetic surveillance of first- and second-line drug-resistant isolates of *Mycobacterium tuberculosis* in Peru

**DOI:** 10.1371/journal.pone.0352881

**Published:** 2026-07-09

**Authors:** David Santos-Lázaro, Aiko N. Vigo, Vidia V. Cotrina, Sandra Villar, Alexis Medina, Cynthia Julcapoma, Edson Pacheco, Miriam J. Alarcón, Zully M. Puyén

**Affiliations:** 1 Laboratorio de Referencia Nacional de Micobacterias, Instituto Nacional de Salud, Lima, Perú; 2 Escuela de Medicina, Universidad Peruana de Ciencias Aplicadas, Lima, Perú; St Petersburg Pasteur Institute, RUSSIAN FEDERATION

## Abstract

Tuberculosis (TB) remains a critical public health crisis in Peru, which bears the highest multidrug-resistant TB (MDR-TB) burden in the Americas. The objective of this study was to conduct a nationwide genetic surveillance of first- and second-line drug-resistant *Mycobacterium tuberculosis* isolates in Peru to characterize their structural and geographic distribution using a multilocus genotype approach. We analyzed 38,339 valid results from first- and second-line line probe assays (LPA 1L, GenoType MTBDR*plus* v2; and LPA 2L, GenoType MTBDR*sl* v2) routinely collected between 2019 and 2022 by the Peruvian National Institute of Health. First-line (G1) and second-line (G2) multilocus genotypes were generated by concatenating specific hybridization banding patterns across resistance-associated loci. Additionally, targeted next-generation sequencing was performed on a stratified proportional sample of 170 isolates exhibiting “inferred” resistance genotypes, defined by a missing wild-type signal without a corresponding mutant probe, to elucidate their underlying mutational profiles. Resistance genotypes were identified in 13.5% and 11.8% of LPA 1L and LPA 2L results, respectively. The genotypic landscape was highly concentrated: 13 dominant G1 genotypes and 19 dominant G2 genotypes accounted for over 85% of their respective resistant cohorts. Structurally, these dominant genotypes mainly carried globally successful, canonical mutations (e.g., *katG* S315T1, *rpoB* S450L). Geographically, dominant genotypes were heavily consolidated within the hyper-endemic central coastal axis (Lima, Callao, and Ica) and the Amazonian department of Ucayali, maintaining highly stable temporal frequencies. Conversely, heteroresistance and complex multi-mutational patterns were exclusively restricted to low-frequency rare and orphan genotypes. Targeted sequencing of inferred patterns successfully expanded the national catalog of resistance variants, identifying non-canonical determinants (e.g., *inhA* g-17t, *gyrB* T500N) and preventing false-positive resistance calls driven by synonymous mutations. This large-scale mapping demonstrates that Peru’s drug-resistant TB epidemic is characterized by the widespread consolidation of a restricted number of canonical multilocus genotypes. Integrating targeted sequencing with routine molecular screening is essential to resolve unclassified hybridization patterns and optimize epidemiological management in high-burden settings.

## Introduction

Tuberculosis (TB), caused by the bacillus *Mycobacterium tuberculosis* (MTB), remains one of the most significant preventable and curable infectious diseases, yet it continues to cause high morbidity and mortality worldwide. In 2024, an estimated of 10.7 million people globally fell ill with TB; however, only 8.3 million people were reported as newly diagnosed, highlighting a persistent gap in global case detection [1].

Drug-resistant TB poses a persistent threat to global public health. Multidrug-resistant TB (MDR-TB), defined as TB caused by MTB isolates resistant to at least isoniazid and rifampicin, is particularly concerning. Additionally, pre-extensively drug-resistant TB (Pre-XDR-TB, MDR or rifampicin-resistant isolates with additional resistance to levofloxacin or moxifloxacin), and extensively drug-resistant TB (XDR-TB, Pre-XDR isolates with further resistance to bedaquiline or linezolid) are even more complex. These resistant forms require very expensive, prolonged, less effective treatments, leading to higher morbidity, toxicity, relapses rates and poor clinical outcomes [2,3].

For years, Peru has been among the 30 countries with the highest MDR-TB burden globally and ranks first in the Americas [2]. Although drug-resistant TB is present in all 24 departments of the country, it is heavily concentrated in the department of Lima and the Constitutional Province of Callao, which together account for most cases nationwide. In alignment with World Health Organization (WHO) recommendations, Peru has implemented line probe assays (LPA) for the molecular diagnosis of TB and detection of resistance to the key first- and second-line drugs [4]. Additionally, the national TB health network ensures that samples from across the country are analyzed using this molecular test, either from sputum samples or solid MTB isolates.

The surveillance of mutations associated with drug resistance is crucial for controlling TB, reducing transmission, improving molecular diagnostics, and strengthening public health strategies. Previous studies in Peru have assessed the genetic diversity of resistance-associated mutations, but they have often focused on specific first- or second-line drugs, been limited to certain geographic areas, or analyzed only particular drug-resistant TB forms [5–8]. Moreover, most of these studies have examined individual mutations in isolation rather than integrating resistance-associated mutations across multiple drug-related genes. In contrast, the use of multilocus resistance genotypes (defined as specific combinations of co-occurring mutations across different resistance-associated genes) allows for a more comprehensive assessment of genetic diversity. By analyzing these genotypes, we can move beyond isolated mutation studies to an integrated approach that captures the broader genetic landscape of resistance [9,10]. This approach not only facilitates the detection of frequent variants within a given geographic region but also enhances our ability to identify emerging low-frequency mutations, ultimately improving our understanding of resistance dynamics at a national level [11].

In this retrospective study, we conducted a nationwide genetic surveillance of first- and second-line drug-resistant *Mycobacterium tuberculosis* isolates in Peru from 2019 to 2022. This surveillance was performed using a multilocus genotype approach to characterize the structural and geographic distribution of resistant strains across the country. Furthermore, we integrated targeted sequencing to elucidate the specific mutational profiles underpinning inferred resistance patterns. By anchoring our analysis in comprehensive, laboratory-based national surveillance, this study provides a high-resolution mapping of the circulating molecular diversity, offering critical baseline data to optimize diagnostic strategies and strengthen tuberculosis control efforts in high-burden settings.

## Materials and methods

### Study setting

This retrospective, cross-sectional study was conducted using routine molecular drug susceptibility testing records maintained at the National Reference Laboratory for Mycobacteria (LRNM; Laboratorio de Referencia Nacional de Micobacterias) of the Peruvian National Institute of Health (INS; Instituto Nacional de Salud). In October 2023, we accessed the complete LRNM-INS database of historical results obtained nationwide between January 1, 2019, and December 31, 2022. The unit of analysis was the *Mycobacterium tuberculosis* isolate and no clinical data were collected.

During this period, GenoType MTBDR*plus* v2 first-line LPA (LPA 1L), used to detect resistance to rifampicin and isoniazid, was performed across four nodes nationwide: three local reference laboratories located in Central, Southern, and Eastern Lima, and the LRNM-INS. While the three local laboratories processed samples strictly from their respective jurisdictions, the LRNM-INS maintained comprehensive national coverage, receiving samples from all 24 departments countrywide. This network structure resulted in a shared testing workflow within the Lima Metropolitan Area; while the entirety of the Constitutional Province of Callao and Northern Lima relied solely on the LRNM-INS, cases from Central, Eastern, and Southern Lima were processed both locally by their local laboratories and centrally by the LRNM-INS. In contrast, GenoType MTBDR*sl* v2 second-line LPA (LPA 2L), used to detect resistance to fluoroquinolones and second-line injectables, was performed exclusively at the LRNM-INS.

As a result of this network architecture, the LRNM-INS registry captured approximately 45% of all LPA 1L tests conducted nationally and 100% of all valid LPA 2L tests. Although the national diagnostic algorithm indicated that LPA 2L was reserved for first-line resistant cases, the centralized LRNM-INS database naturally contains a higher volume of LPA 2L records relative to its own LPA 1L resistant entries. This discrepancy is accounted for by the systematic incorporation of referred data from the three local Lima laboratories, which required only second-line centralized processing at the LRNM-INS (S1 Fig).

### Selection and interpretation of LPA strips

All LPA strips archived at the LRNM-INS were initially screened according to predefined inclusion criteria: (i) presence of all internal control bands (conjugate and amplification controls) and the *TUB* band, confirming the presence of the MTB complex; (ii) banding patterns consistent with the manufacturer’s quality control guidelines (Hain Lifescience GmbH, Nehren, Germany). Duplicate laboratory results of the same sample were subsequently excluded, ensuring that each included result represented a distinct testing event. Genotypic resistance patterns for second-line drugs were determined for levofloxacin, moxifloxacin and amikacin, in accordance with updated WHO guideline [4].

LPA results were classified into three categories: i) resistance not detected, defined as the presence of all *wild-type* (WT) bands and absence of all *mutant* (MUT) bands; ii) resistance detected, defined as the presence of one or more MUT bands; and iii) resistance inferred, defined as the absence of one or more WT bands in the absence of corresponding MUT bands. Specific mutations associated with each MUT probe were identified following the manufacturer’s instructions. Mutations conferring resistance to isoniazid and moxifloxacin were further stratified into low- and high-level resistance categories, as recommended by WHO [4]. Both homoresistance (absence of WT bands with presence of the corresponding MUT bands) and heteroresistance (simultaneous presence of WT and corresponding MUT bands) were considered.

### Determination of drug-resistant genotypes

Resistance patterns were analyzed to identified drug-resistant genotypes using GeneAlEx v6.503 [12]. Resistant genotypes were defined as unique combinations of resistance-associated mutations in the target genes interrogated by LPAs 1L and 2L, separately. Hybridization patterns from DNA probes were converted into binary matrices indicating the presence or absence of specific bands. First-line (G1) and second-line (G2) resistance genotypes were generated by concatenating banding patterns from resistance-associated loci only. During matrix compilation, the software assigned consecutive nominal identifiers based on initial detection order (e.g., G1-1, G1-2). Genotypes were classified based on their relative frequencies (n) as follows: common (n > 5%), less common (1% ≤ n ≤ 5%), rare (n < 1%; excluding n = 1) and orphan genotypes (n = 1) [13].

### Targeted sequencing

A stratified purposeful sampling strategy was employed to select isolates for targeted sequencing, focusing specifically on isolates exhibiting inferred resistance genotypes. From a total universe of 584 available isolates with inferred genotypes, a sample of 170 isolates was selected for sequencing due to reagent availability and sequencing capacity constraints. To ensure representativeness, these 170 isolates were selected using a stratified proportional sampling design that preserved the national database ratio of first-line (485 isolates in the universe) and second-line (99 isolates in the universe) inferred genotypes. This sample size provides a 95% confidence level with a margin of error of approximately 6.3%, assuming maximum variability (*p* = 0.5), ensuring the representativeness of circulating strains with these inferred resistance genotypes.

Genomic DNA was extracted from cryopreserved MTB isolates using the Presto Mini gDNA Bacteria Kit (Geneaid Biotech, Taiwan), following the manufacturer’s protocol for Gram-positive bacteria. Multiplex PCR of *rpoB*, *inhA*, *gyrA*, *gyrB* and *rrs* genes was performed with *in-house* primers (S1 Table) in a 25 uL reaction (5 uL of 5xTransStart FastPfu Buffer, 2 uL of 2.5 mM of each dNTPs, 5 uL of primer Mix, 0.5 uL of hot-start high-fidelity TransStart FastPfu DNA Polymerase, 7.5 uL molecular biology-grade water, and 5 uL template DNA). Cycling conditions on a Gradient PCR Optima 96 system included: 95°C for 2 minutes; 40 cycles of 95°C for 20 seconds, 65°C for 30 seconds, and 72°C for 80 seconds; with a 5 minutes of final extension at 72°C. Amplicons were sequenced using Next-Generation Sequencing on the Illumina MiSeq platform (2 × 150 bp paired-end reads). Raw sequencing data are available in the NCBI Sequence Read Archive (BioProject PRJNA1265395).

### Statistical analysis

Temporal changes in the frequencies of common G1 and G2 genotypes were evaluated using the chi-square (χ²) test implemented in R v4.4.2 [14]. To account for potential bias due to unequal sample sizes across years, absolute genotype counts were normalized using the year with the lowest number of analyzed isolates as reference. A two-sided p-value < 0.05 was considered statistically significant.

### Ethic statement

This study was conducted as part of a research project approved by the Institutional Research Ethics Committee of the INS (approval code: OC-028–21). The study was carried out with the authorization of the INS National Reference Laboratory for Mycobacteria, using anonymized data and bacterial isolates collected as part of routine diagnostic testing at the national reference laboratory between 2019 and 2022. No personally identifiable information was collected, and all samples were anonymized using unique study-specific codes. Informed consent was not required due to the retrospective use of de-identified, routinely collected data.

## Results

### Sample characteristics and molecular resistant profiles

A total of 38,339 valid LPA results were analyzed, including 27,519 from LPA 1L and 10,820 from LPA 2L. Resistance-associated genotypes were detected in 3,726 (13.5%) LPA 1L results and 1,278 (11.8%) LPA 2L results. 90% (3,379/3,726) of the LPA 1L–resistant isolates yielded valid LPA 2L results at the LRNM-INS. However, a substantial proportion of these 3,379 isolates showed fully susceptible LPA 2L patterns, which precluded meaningful genotype-based analyses for second-line resistance. Consequently, genotypes were analyzed independently for LPA 1L and LPA 2L. Geographically, resistant isolates for LPA 1L were reported across all 24 departments of Peru, with 70.5% (2,625/3,726) of the entries concentrated in Lima, Callao, Ica, Ucayali, Ancash, Junín and Loreto. For LPA 2L, Lima, Callao and Ica accounted for 79.3% (1,013/1,278) of resistant isolates, whereas no resistant results were observed in Pasco, Apurímac, or Huancavelica (S2 Table).

Regarding genetic resistance among the LPA 1L cohort, rifampicin resistance was detected in 2,339 isolates, comprising 1,977 MDR and 362 rifampicin mono-resistant [RMR] entries. At nucleotide level, this resistance was dominated by canonical substitutions within the *rpoB* rifampicin resistance-determining region (RRDR), with S450L emerging as the most prevalent variant followed by D435V. On the other hand, isoniazid resistance was identified in 1,387 isoniazid mono-resistant (HMR) isolates. This resistance was mainly explained by mutations in *katG* gene and *inhA* promoter region, with most resistant isolates harboring *katG* S315T1, *inhA* c-15t or both. High-level resistance predominated over low-level variants. Overall, MDR-TB profiles accounted for 59% of all isoniazid-resistant and 85% of rifampicin-resistant isolates.

In contrast, within the LPA 2L cohort, 309 isolates showed simultaneous resistance to levofloxacin/moxifloxacin and amikacin (Lfx/Mfx-Am R), 692 exhibited levofloxacin/moxifloxacin resistance only (Lfx/Mfx R) and 277 showed amikacin resistant only (Am R). Levofloxacin/moxifloxacin resistance was mainly driven by *gyrA* mutations concentrated at codons 94 and 90, whereas *gyrB* mutations contributed marginally. These variants were associated with both high- and low-level moxifloxacin resistance (low-level resistance being slightly more frequent). Meanwhile, amikacin resistance was overwhelming associated with the non-coding *rrs* a1401g mutation (S3 Table).

### Drug-resistant genotypes

Regarding the first-line cohort, a total of 96 G1 genotypes were identified, displaying a high concentration of resistance within a limited number of molecular patterns. Specifically, 13 dominant genotypes (comprising six common and seven less common) accounted for 88.9% (3,313/3,726) of all first-line resistant isolates, encompassing the principal MDR, HMR and RMR profiles (Table 1). In contrast, the remaining first-line resistant isolates were distributed among 51 rare and 32 orphan genotypes. While these low-frequency genotypes collectively represented slightly over 10% of the cohort, they exhibited substantially greater diversity in resistance patterns and mutational combinations compared to the dominant variants. Notably, while dominant genotypes exclusively carried homoresistant mutations, heteroresistance was restricted to these low-frequency categories (occurring in 19 rare and 12 orphan genotypes) and accounted for 2.8% (103/3726) of isolates. Whitin these heteroresistant configurations, mutations conferring resistance to isoniazid occurred at least twice as frequently as those for rifampicin. Furthermore, double mutations were uncommon and restricted to the *rpoB* gene, occurring exclusively among rare and orphan genotypes across 10 isolates, seven of which were heteroresistant (S4 Table).

**Table 1:** Dominant first-line MTB genotypes identified by line probe assays in Peru.

Class	Code	No.	*rpoB*	*katG*	*inhA*	Profile
Common	G1-57	750	S450L	S315T1	–	MDR
	G1-70	532	–	S315T1	–	HMR
	G1-73	474	–	–	c-15t	HMR
	G1-59	444	S450L	–	c-15t	MDR
	G1-18	379	D435V	S315T1	–	MDR
	G1-72	214	–	–	ΔWT1	HMR
Less common	G1-60	154	S450L	–	–	RMR
	G1-68	95	–	S315T1	c-15t	HMR
	G1-24	73	ΔWT3	–	–	RMR
	G1-11	61	ΔWT3,4	S315T1	–	MDR
	G1-43	53	ΔWT8	S315T1	–	MDR
	G1-48	45	ΔWT8	–	–	RMR
	G1-29	39	ΔWT7	S315T1	–	MDR

G1, first-line genotype; MDR, multidrug-resistant; HMR, isoniazid mono-resistant; RMR, rifampicin mono-resistant; ΔWT, absence of *wild type* band. No., number of isolates with the respective genotype.

A highly consistent architectural pattern was observed within the second-line cohort, where 101 G2 genotypes were identified. Resistance was highly concentrated in 19 dominant genotypes (seven common and twelve less common) accounting for 85.4% (1,092/1,278) of the isolates and capturing the most frequent Lfx/Mfx-Am R, Lfx/Mfx R, and Am R profiles (Table 2). Conversely, the remaining second-line resistant isolates were composed of 35 rare and 47 orphan genotypes, which displayed a markedly higher diversity of mutational combinations than the dominant counterparts. Heteroresistance was confined to three less common, 18 rare, and 26 orphan genotypes, and was detected at least twice as often for levofloxacin or moxifloxacin compared to amikacin. Additionally, advanced mutational complexity, manifested as double and triple mutations, were exclusively detected within the *gyrA* gene and occurred only among rare and orphan genotypes. This complex genetic signature encompassed 39 isolates, of which 19 were heteroresistant (S5 Table).

**Table 2:** Dominant second-line MTB genotypes identified by line probe assays in Peru.

Class	Code	No.	*gyrA*	*gyrB*	*rrs*	*eis*	Profile
Common	G2-66	217	–	–	a1401g	–	Am R
	G2-16	150	A90V	–	–	–	Lfx/Mfx R
	G2-35	131	D94G	–	–	–	Lfx/Mfx R
	G2-52	105	D94A	–	–	–	Lfx/Mfx R
	G2-41	81	D94N/Y	–	a1401g	–	Lfx/Mfx-Am R
	G2-43	66	D94N/Y	–	–	–	Lfx/Mfx R
	G2-30	64	D94G	–	a1401g	–	Lfx/Mfx-Am R
Less common	G2-13	42	A90V	–	a1401g	–	Lfx/Mfx-Am R
	G2-60	35	–	ΔWT	–	–	Lfx/Mfx R
	G2-9	32	S91P.	–	–	–	Lfx/Mfx R
	G2-77	26	D94G	–	–	–	Lfx/Mfx R
	G2-2	22	ΔWT1	–	–	–	Lfx/Mfx R
	G2-59	22	–	ΔWT	a1401g	–	Lfx/Mfx-Am R
	G2-91	21	A90V	–	–	–	Lfx/Mfx R
	G2-65	20	–	–	ΔWT1	–	Am R
	G2-70	18	–	–	a1401g	–	Am R
	G2-50	14	D94A	–	a1401g	–	Lfx/Mfx-Am R
	G2-64	13	–	N538D	–	–	Lfx/Mfx R
	G2-67	13	–	–	–	c-14t	Am R

G2, second-line genotype; Lfx/Mfx-Am R, levofloxacin/moxifloxacin and amikacin-resistant; Lfx/Mfx R, levofloxacin/moxifloxacin-resistant; Am R, amikacin-resistant; ΔWT, absence of *wild type* band. No., number of isolates with the respective genotype.

### Mutation patterns of inferred genotypes

A total of 170 samples were successfully sequenced, including 141 from G1 and 29 from G2 genotypes. Across both groups, most inferred genotypes displayed multiple mutations at comparable frequencies, although a subset was dominated by a single, recurrent mutation.

Among G1 genotypes, several patterns were driven by a predominant mutation. The G1-72 genotype was largely explained by the *inhA* g-17t mutation, whereas G1-24 was exclusively associated with the synonymous *rpoB* F433F mutation. Other G1 genotypes exhibited greater mutational heterogeneity within the *rpoB* gene.

Similarly, among G2 genotypes, inferred resistance patterns were frequently dominated by a single mutation. The G2-60 genotype was mainly associated with *gyrB* T500N, G2-2 with *gyrA* G88C, and G2-59 with *gyrB* E501D, indicating consistent mutation-genotype associations despite lower overall frequencies.

A small number of isolates lacked detectable mutations within the sequenced regions. These included isolated samples within G1-72, G1-11 and G2-2, as well as a substantial proportion of G1-48 isolates and all sequenced isolates belonging to G2-65. With one exception, all isolates carried a single mutation with an allelic frequency of at least 98%. The exception was a single G1-29 isolate, which harbored two *rpoB* mutations at different proportions (H445R [40%] and H445L [60%]), consistent with intraisolate heterogeneity.

Although inferred genotypes were expected to yield resistant profiles distinct from directly detected mutations, a limited number of isolates nonetheless harbored directly detectable resistance mutations, including *inhA* c-15t, *rpoB* D435V, and *rpoB* S450L (Table 3).

**Table 3:** Mutational profiles of first- and second-line drug-resistant MTB genotypes with inferred resistance identified in Peru.

Genotype	No.	Mutation (no.)	
**First Line**		**Rifampicin**	**Isoniazid**
**G1-72**	**69**		***inhA* ΔWT1**
			g-17t (67)
			c-15t, g-17c (1)
			No mutation (1)
**G1-24**	**24**	***rpoB* ΔWT3**	
		F433F (24)	
**G1-11**	**17**	***rpoB* ΔWT3,4**	***katG* S315T1**
		D435F (5)	S315T1 (17)
		D435Y (9)	
		D435V; N437D (1)	
		F433S; D435Y (1)	
		No mutation (1)	
**G1-43**	**12**	***rpoB* ΔWT8**	***katG* S315T1**
		L452P (1)	S315T1 (12)
		S450L* (4)	
		S450F (4)	
		S450W (3)	
**G1-48**	**8**	***rpoB* ΔWT8**	
		L449Q (1)	
		L452P (2)	
		S450W (1)	
		No mutation (4)	
**G1-29**	**11**	***rpoB* ΔWT7**	***katG* S315T1**
		H445R; R448L (3)	S315T1 (11)
		H445R†; H445L† (1)	
		L430R; H445P (1)	
		H445N (3)	
		H445L (3)	
**Second Line**		**Levofloxacin/Moxifloxacin**	**Amikacin**
**G2-60**	**13**	***gyrB* ΔWT**	
		N499K (2)	
		E501D (1)	
		T500A (1)	
		T500N (9)	
**G2-2**	**7**	***gyrA* ΔWT1**	
		G88C (6)	
		No mutation (1)	
**G2-59**	**7**	***gyrB* ΔWT**	***rrs* a1401g**
		N499I (1)	a1401g (7)
		E501D (6)	
**G2-65**	**2**		***rrs* ΔWT1**
			No mutation (2)

The numbers in parentheses represent the number of isolates in which each specific mutation was detected through sequencing. G1, first-line genotype; G2, second-line genotype; ΔWT, absence of *wild type* band. No., number of isolates with the respective genotype.

* mutations that should have been detected directly.† intraisolate heterogeneity.

### Spatiotemporal distribution of common genotypes

**Fig 1:**
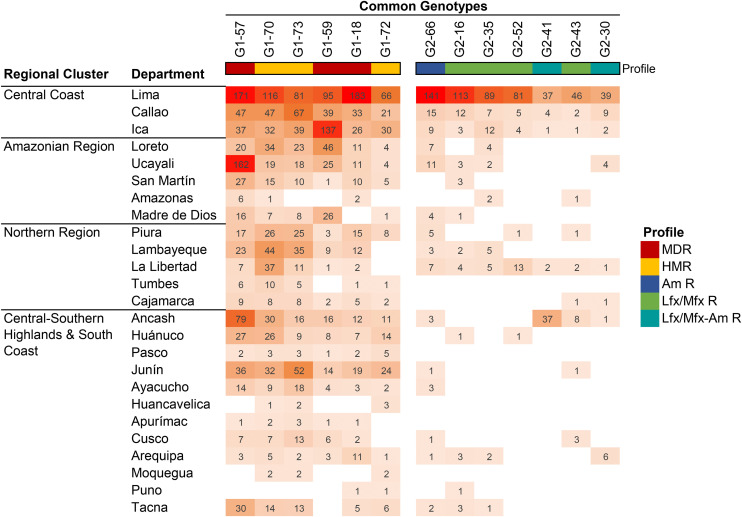
National distribution of common drug-resistant MTB genotypes identified by first- and second-line line probe assays. The heatmap shows the distribution of common genotypes detected by GenoType MTBDR*plus* v2 and MTBDR*sl* v2 assays across Peru, operationally grouped for analytical purposes to highlight regional density clustering. Numbers in the cells indicate sample counts, with darker colors showing higher isolates concentration. Resistant profiles were classified by first-line resistance as MDR (multidrug-resistant) and HMR (isoniazid mono-resistant), and by second-line resistance as Lfx/Mfx-Am R (levofloxacin/moxifloxacin and amikacin-resistant), Lfx/Mfx R (levofloxacin/moxifloxacin-resistant), and Am R (amikacin-resistant). G1 and G2 refer to first- and second-line genotypes, respectively.

Regarding temporal trends, the absolute frequencies of common G1 and G2 genotypes remained highly stable across the study period. Rare exceptions to this temporal stability included a significant increase in G1-73 during 2022 (*p* = 0.002), a transient increased in G1-59 in 2020 (*p* = 0.037), and a marked decline in both 2020 and 2022 for G2-30 (*p* = 0.001) (S6 Table). Geographically, the distribution of the common genotypes revealed distinct epidemiological patterns across the country (Fig 1). G1 genotypes exhibited a hyper-endemic core concentrated in the central coastal region (Lima, Callao, and Ica) and the jungle department of Ucayali, where the MDR genotype G1-57 demonstrated a disproportionately high localized burden (n = 162). In contrast, departments in the southern highlands and coast displayed markedly lower sample volumes. Huancavelica and Moquegua exhibited a complete polarization toward HMR genotypes.

A much more restricted and fragmented geographic landscape emerged for G2 genotypes. Resistance was overwhelmingly driven by the amikacin-resistant G2-66 and the three Lfx/Mfx R genotypes (G2-16, G2-35, and G2-52). Outside of Lima, the dispersal of second-line genotypes dropped abruptly, appearing only sporadically as isolated clusters, while remaining entirely undetected across five departments (Tumbes, Pasco, Huancavelica, Moquegua, and Apurimac) (Fig 1).

## Discussion

TB remains a critical public health emergency in Peru, a nation consistently ranked among the 30 countries with the highest burden of MDR-TB worldwide [2,15]. A major novelty of this study lies in its genotype-based analytical approach. While traditional surveillance often evaluates resistance-conferring mutations in isolation, characterizing complete molecular genotypes, defined by specific combinatorial hybridization patterns, allows for a higher-resolution alignment of co-occurring mutations, improving the overall mapping of drug-resistant strains. Applying this framework, our findings underscore a high national frequency of MDR genotypes alongside a substantial, persistent burden of HMR. Notably, the proportion of HMR-TB isolates consistently exceeded that of RMR-TB, mirroring a trend reported globally [10,16,17]. Failing to systematically detect these HMR genotypes leads to their misclassification as drug-susceptible, resulting in suboptimal standard treatment regimens that drive therapeutic failures and progression to MDR-TB or resistance to additional drugs [18–21].

The genotypic architecture of circulating MTB strains in Peru was characterized by a high concentration of resistance within a limited number of genotypes, with a few dominant genotypes accounting for over 85% of both first- and second-line resistant isolates. Crucially, the molecular composition of these dominant genotypes was driven by canonical mutations, such as *rpoB* S450L and D435V defining MDR genotypes, *katG* S315T1 driving isoniazid resistance, and *gyrA* variants at codons 90 and 94 driving fluoroquinolone resistance, all of which have been extensively documented as the globally most prevalent resistance determinants [22–25]. The fact that these specific globally successful substitutions underpin the dominant Peruvian genotypes explains their broad geographical dispersal, particularly concentrated within the hyper-endemic central coastal axis of Lima, Callao, and Ica. While the analysis of drug-target loci alone cannot definitively differentiate clonal transmission from parallel independent evolution, this highly asymmetrical distribution of highly fit canonical mutations in the high-density capital region strongly points toward a scenario where sustained transmission plays a important role. Under the intense selective pressure of the urban setting, these successful alleles likely perpetuate throughout the community, as evidenced in previous studies [8,22,26]. Conversely, the vast mutational diversity and alternative non-canonical substitutions observed among rare and orphan genotypes reflect sporadic, independent emergence events under localized selective pressures [27].

Within this genotype framework, the integration of targeted sequencing proved essential to resolve the diagnostic ambiguities inherent to LPAs. While dominant genotypes were overwhelmingly defined by canonical, directly detected mutations, relying exclusively on LPA hybridization for less common genotypes often yielded inferred results that complicate clinical management. The genetic characterization of LPA 1L resistance in Peru was conducted several years ago, revealing a high proportion of isolates with inferred resistance [10]. In the present study, we expanded upon these findings by incorporating LPA 2L results and applying targeted sequencing to identified specific resistance-associated mutations, in accordance with WHO-endorsed criteria [28]. Sequencing successfully elucidated the genetic basis of several key inferred genotypes. For example, we determined that specific inferred HMR genotypes were predominantly driven by the *inhA* g-17t mutation, a variant only recently formalized in the second version of WHO mutation catalog [8,29,30]*.* Similarly, sequencing identified non-canonical *gyrB* substitutions (N499K, E501D, T500A, T500N, and N499I) responsible for inferred low-level fluoroquinolone resistance [29,31,32]. Most importantly, this high-resolution approach safeguarded against false-positive resistance calls by revealing that certain inferred RMR genotypes actually harbored the synonymous mutation *rpoB* F433F, a variant unlinked to phenotypic resistance [33,34]. Consequently, apparent resistance driven solely by the lack of wild-type probe hybridization may often reflect method-specific artifacts or silent mutations rather than true therapeutic threats [35–37].

This genotype-based approach also provided critical insights into the genetic basis of heteroresistance. Notably, the coexistence of resistant and susceptible bacterial subpopulations was restricted to low-frequency rare or orphan genotypes. Overall, these heteroresistance rates (2.8% for first-line and 11.7% for second-line drugs) align with figures reported in other high drug-resistant TB burden countries [38–41]. Biologically, the strict confinement of heteroresistance to minority genotypes strongly supports the model that represents an early stage in the evolution toward more complex drug-resistant forms, potentially marking transitional states before the fixation of resistance, which can negatively impact TB treatment outcomes [39,40,42]. Interestingly, the highest levels of heteroresistance in our cohort were detected in genes associated with isoniazid and levofloxacin resistance, contrasting with previous global reports where rifampin and fluoroquinolones heteroresistance predominates [40,43], further underscoring the intense selective pressures acting upon isoniazid pathways in Peru.

A primary strength of this study is its unprecedented national representativeness. By analyzing the total universe of tests from the LRNM-INS, we captured approximately 45% of all national LPA 1L tests and 100% of the valid LPA 2L registries generated in Peru between 2019 and 2022, providing a comprehensive mapping for second-line resistance. Additionally, for LPA 1L, our dataset covers all 24 departments, including the highest-burden areas of Lima and Callao, ensuring that the findings accurately reflect the epidemiological landscape of drug-resistant TB in the country. Likewise, participation of the LRNM in annual external performance evaluations for LPA methodology ensures the reliability and consistency of the results. However, some limitations should be noted. Genetic characterization was limited to the regions interrogated by both LPAs, and mutations in additional genes associated with drug resistance were not assessed. Consequently, results reflect the distribution of resistance-associated genotypes among tested isolates rather than the full spectrum of resistance determinants. In addition, the unit of analysis was the MTB isolate rather than the individual patient. Although duplicate laboratory results of the same sample were excluded, there is a potential for inclusion of isolates from the same patient collected in different calendar years, reflecting routine programmatic testing. Furthermore, no clinical data was collected for this study, including patient treatment history. Therefore, the reported frequencies do not represent patient-level prevalence. Future studies that include clinical information are needed to assess the relationship between resistant genotypes and patient treatment history. Despite these constraints, inclusion of the most prevalent resistance-associated markers in Peru and globally captures most of the clinically relevant genetic diversity within key resistance loci of circulating MTB strains.

In conclusion, this study establishes a nationwide framework for the genetic surveillance of first- and second-line drug-resistant *Mycobacterium tuberculosis* isolates in Peru. Our findings demonstrate that the national resistance landscape is driven by highly stable, dominant multilocus genotypes heavily consolidated within the metropolitan coastal axis. Furthermore, integrating targeted sequencing with routine molecular screening proved essential to resolve the specific mutational profiles, and capture non-canonical resistance determinants, providing critical baseline data to guide the future optimization of diagnostic strategies and strengthen tuberculosis control efforts in high-burden settings.

## Supporting information

S1 Fig Workflow of the national laboratory network for LPA screening of drug-resistant *Mycobacterium tuberculosis* isolates. (PDF)

S1 Table Genetic sequences of *in house* designed primers used for targeted Next Generation Sequencing of resistant genotypes with inferred resistance. (PDF)

S2 Table Number of valid results obtained for first- and second-line line probe assays and its location distribution across the entire country. (PDF)

S3 Table Frequency of mutations conferring drug resistance in *Mycobacterium tuberculosis* identified by first- and second-line line probe assays. (PDF)

S4 Table Complete set of first-line resistant genotypes obtained through GenoType MTBDR*plus* v2. (PDF)

S5 Table Complete set of second-line resistant genotypes obtained through GenoType MTBDR*sl* v2. (PDF)

S6 Table Temporal variation in the frequency of common drug-resistant genotypes of *Mycobacterium tuberculosis* in Peru, 2019–2022, based on observed and adjusted counts. (PDF)
