# Supplementary figures and images for: Genetic surveillance of first- and second-line drug-resistant isolates of Mycobacterium tuberculosis in Peru
Source: PLoS One. 2026 Jul 9;21(7):e0352881. doi: 10.1371/journal.pone.0352881 (PMC13349105; doi:10.1371/journal.pone.0352881)

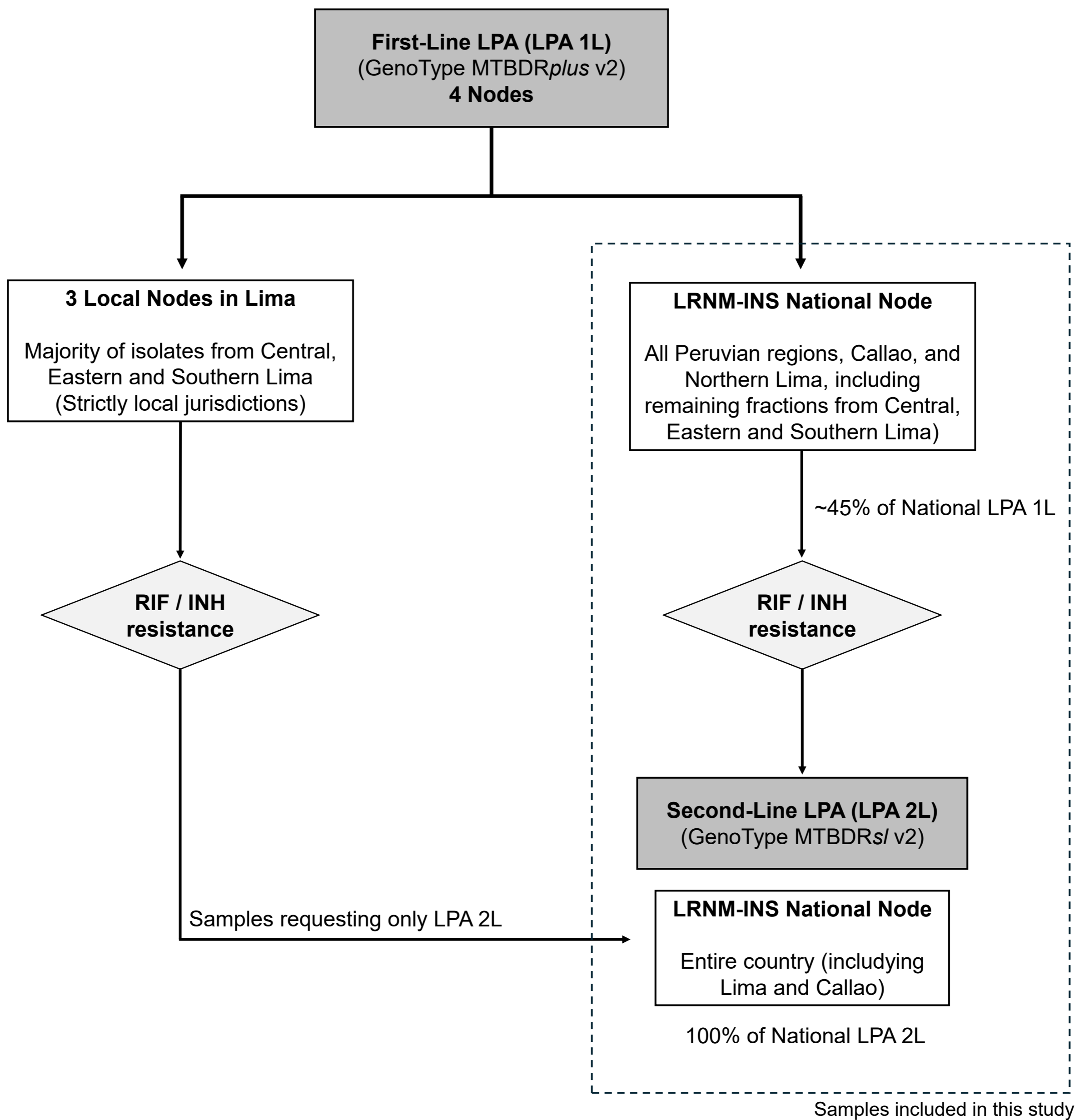

Supplement: S1 Fig — (PDF) [file pone.0352881.s001.pdf]
