# Supplementary material for: Genetic surveillance of first- and second-line drug-resistant isolates of Mycobacterium tuberculosis in Peru
Source: PLoS One. 2026 Jul 9;21(7):e0352881. doi: 10.1371/journal.pone.0352881 (PMC13349105; doi:10.1371/journal.pone.0352881)
Supplement: S1 Table — (PDF) [file pone.0352881.s002.pdf]

S1 Table. Genetic sequences of *in house* designed primers used for targeted Next Generation Sequencing of resistant genoypes with inferred resistance.

Primer	Sequence
*gyrB*_F	5'-ACCGCAAGCTACTGAAGGACAA-3’
*gyrB*_R	5'-CCCTTGTACCGCTGAATGC-3’
*gyrA*_F	5'-ACCCTGCGTTCGATTGCA-3’
gyrA_R	5'-ACGGCAACTCGGTGATCAC-3’
*rpoB*_F	5'-ATGTCGGCGAGCCCATCA-3’
*rpoB*_R	5'-GTGGCCACCGACACCATCT-3’
*inhA*_F	5'-TAACCCCAGTGCGAAAGTTC-3’
*inhA*_R	5'-CCGGAGATATAGCTCGCATCCT-3’
*katG*_F	5'-GACCCATGTCTCGGTGGAT-3’
*katG*_R	5'-GTGGCCGGTCAAGAAGAAGTAC-3’
*rrs*_F	5'-TTCTCTCGGATTGACGGTAGGT-3’
*rrs*_R	5'-GTTTTCGTGGTGCTCCTTAGA-3’
