# Supplementary material for: Genetic surveillance of first- and second-line drug-resistant isolates of Mycobacterium tuberculosis in Peru
Source: PLoS One. 2026 Jul 9;21(7):e0352881. doi: 10.1371/journal.pone.0352881 (PMC13349105; doi:10.1371/journal.pone.0352881)
Supplement: S2 Table — (PDF) [file pone.0352881.s003.pdf]

S2 Table. Number of valid results obtained for first- and second-line line probe assays and its location distribution across the entire country.

Location	LPA 1L No. (%)	LPA 2L No. (%)	Both LPAs No. (%)
Lima	948 (25.4)	874 (68.4)	1,822 (36.4)
Ica	366 (9.8)	59 (4.6)	425 (8.5)
Callao	311 (8.3)	80 (6.3)	391 (7.8)
Ucayali	317 (8.5)	22 (1.7)	339 (6.8)
Ancash	244 (6.5)	63 (4.9)	307 (6.1)
Junín	241 (6.5)	8 (0.6)	249 (5.0)
Loreto	198 (5.3)	21 (1.6)	219 (4.4)
Lambayeque	161 (4.3)	15 (1.2)	176 (3.5)
La Libertad	99 (2.7)	50 (3.9)	149 (3.0)
Huánuco	136 (3.7)	7 (0.5)	143 (2.9)
Piura	119 (3.2)	8 (0.6)	127 (2.5)
Tacna	94 (2.5)	11 (0.9)	105 (2.1)
San Martín	90 (2.4)	7 (0.5)	97 (1.9)
Madre de Dios	72 (1.9)	6 (0.5)	78 (1.6)
Ayacucho	68 (1.8)	6 (0.5)	74 (1.5)
Cusco	43 (1.2)	4 (0.3)	47 (0.9)
Cajamarca	43 (1.2)	3 (0.2)	46 (0.9)
Arequipa	28 (0.8)	16 (1.3)	44 (0.9)
Tumbes	32 (0.9)	3 (0.2)	35 (0.7)
Pasco	20 (0.5)	0 (0.0)	20 (0.4)
Amazonas	13 (0.3)	3 (0.2)	16 (0.3)
Apurímac	9 (0.2)	0 (0.0)	9 (0.2)
Moquegua	8 (0.2)	1 (0.1)	9 (0.2)
Huancavelica	8 (0.2)	0 (0.0)	8 (0.2)
Puno	2 (0.1)	2 (0.2)	4 (0.1)
No information	56 (1.5)	9 (0.7)	65 (1.3)
**Total**	**3,726 (100)**	**1,278 (100)**	**5,004 (100)**

LPA 1L, first-line line probe assay; LPA 2L, second-line line probe assay.
