# Supplementary material for: Genetic surveillance of first- and second-line drug-resistant isolates of Mycobacterium tuberculosis in Peru
Source: PLoS One. 2026 Jul 9;21(7):e0352881. doi: 10.1371/journal.pone.0352881 (PMC13349105; doi:10.1371/journal.pone.0352881)
Supplement: S3 Table — (PDF) [file pone.0352881.s004.pdf]

S3 Table: Frequency of mutations conferring drug resistance in *Mycobacterium tuberculosis* identified by first- and second-line line probe assays.

Drug	Mutation	No. (%)	Level of resistance*
Rifampicin	rpoB_S450L	1,412 (60.4)	
	rpoB_D435V	413 (17.7)	
	rpoB_ΔWT8	118 (5.0)	
	rpoB_ΔWT3	93 (4.0)	
	rpoB_ΔWT3,4	77 (3.3)	
	rpoB_ΔWT7	60 (2.6)	
	rpoB_H445D	58 (2.5)	
	rpoB_H445Y	58 (2.5)	
	rpoB_ΔWT2	19 (0.8)	
	rpoB_ΔWT2,3	16 (0.7)	
	Others†	15 (0.6)	
Isoniazid	katG_S315T1	1,954 (58.1)	High level
	inhA_c-15t	991 (29.5)	At least low level
	inhA_ΔWT1	233 (6.9)	At least low level
	katG_S315T1, inhA_c-15t	125 (3.7)	High level
	katG_S315T2	16 (0.5)	High level
	inhA_t-8c	13 (0.4)	At least low level
	katG_ΔWT	11 (0.3)	High level
	Others†	21 (0.6)	
Levofloxacin, Moxifloxacin	gyrA_D94G	234 (23.4)	High level
	gyrA_A90V	234 (23.4)	At least low level
	gyrA_D94N/Y	157 (15.7)	High level
	gyrA_D94A	133 (13.3)	At least low level
	gyrB_ΔWT	58 (5.8)	At least low level
	gyrA_S91P	47 (4.7)	At least low level
	gyrA_ΔWT1	28 (2.8)	At least low level
	gyrA_ΔWT3	23 (2.3)	At least low level
	gyrB_N538D	19 (1.9)	At least low level
	gyrA_D94H	11 (1.1)	High level
	Others†	57 (5.7)	
Amikacin	rrs_a1401g	516 (86.1)	
	rrs_ΔWT1	28 (4.7)	
	eis_c-14t	24 (4.0)	
	Others†	31 (5.2)	

*Resistance levels only for isoniazid and moxifloxacin according the latest WHO guidelines on interpreting and reporting line probe assays for drug-resistant tuberculosis [1]. †Mutations with absolute frequencies < 10. **No.**: number of isolates carrying the corresponding mutation.

1. Geneva: World Health Organization. Line probe assays for detection of drug-resistant tuberculosis: interpretation and reporting manual for laboratory staff and clinicians. 2022. Available: https://www.who.int/publications/i/item/9789240046665
