# Supplementary material for: Genetic surveillance of first- and second-line drug-resistant isolates of Mycobacterium tuberculosis in Peru
Source: PLoS One. 2026 Jul 9;21(7):e0352881. doi: 10.1371/journal.pone.0352881 (PMC13349105; doi:10.1371/journal.pone.0352881)
Supplement: S4 Table — (PDF) [file pone.0352881.s005.pdf]

S4 Table. Complete set of first-line resistant Genotypes obtained through GenoType MTBDR*plus* v2.

Genotype Code	No.	Category	Mutation		
			rpoB	***katG***	***inhA***
G1-1	1	orphan	ΔWT1; ΔWT8	-	c-15t
G1-2	1	orphan	ΔWT1	S315T1	-
G1-3	2	rare	ΔWT1	-	-
G1-4	13	rare	ΔWT2,3	S315T1	-
G1-5	3	rare	ΔWT2,3	-	-
G1-6	1	orphan	ΔWT2	S315T1	-
G1-7	11	rare	ΔWT2	-	c-15t
G1-8	7	rare	ΔWT2	-	-
G1-9	1	orphan	ΔWT3,4; H445D	-	c-15t
G1-10	1	orphan	ΔWT3,4	Δ	-
G1-11	61	less common	ΔWT3,4	S315T1	-
G1-12	2	rare	ΔWT3,4	-	c-15t
G1-13	12	rare	ΔWT3,4	-	-
G1-14	1	orphan	ΔWT3,4	S315T1*	-
G1-15	1	orphan	D435V	ΔWT	-
G1-16	4	rare	D435V	S315T1	c-15t
G1-17	1	orphan	D435V	S315T1	t-8c
G1-18	379	common	D435V	S315T1	-
G1-19	1	orphan	D435V	-	c-15t
G1-20	12	rare	D435V	-	-
G1-21	3	rare	D435V	S315T1*	-
G1-22	1	orphan	ΔWT3; H445D	S315T1	-
G1-23	20	rare	ΔWT3	S315T1	-
G1-24	73	less common	ΔWT3	-	-
G1-25	4	rare	ΔWT3; D435V*	S315T1	-
G1-26	1	orphan	ΔWT4,5	-	-
G1-27	1	orphan	ΔWT5,6	-	c-15t
G1-28	7	rare	ΔWT7	S315T1	c-15t
G1-29	39	less common	ΔWT7	S315T1	-
G1-30	2	rare	ΔWT7	-	ΔWT1
G1-31	5	rare	ΔWT7	-	c-15t
G1-32	7	rare	ΔWT7	-	-
G1-33	3	rare	H445D	ΔWT	-
G1-34	27	rare	H445D	S315T1	-
G1-35	2	rare	H445D	-	ΔWT1
G1-36	4	rare	H445D	-	c-15t
G1-37	17	rare	H445D	-	-
G1-38	1	orphan	H445Y	Δ	-

G1-39	18	rare	H445Y	S315T1	-
G1-40	18	rare	H445Y	-	c-15t
G1-41	19	rare	H445Y	-	-
G1-42	3	rare	ΔWT8	S315T1	c-15t
G1-43	53	less common	ΔWT8	S315T1	-
G1-44	1	orphan	ΔWT8	S315T1	c-15t*
G1-45	1	orphan	ΔWT8	-	Δ
G1-46	1	orphan	ΔWT8	-	ΔWT1
G1-47	10	rare	ΔWT8	-	c-15t
G1-48	45	less common	ΔWT8	-	-
G1-49	1	orphan	ΔWT8	-	c-15t*
G1-50	3	rare	ΔWT8	S315T1*	-
G1-51	1	orphan	S450L	Δ	c-15t
G1-52	1	orphan	S450L	Δ	-
G1-53	1	orphan	S450L	ΔWT	-
G1-54	11	rare	S450L	S315T2	-
G1-55	1	orphan	S450L	S315T1	ΔWT1
G1-56	4	rare	S450L	S315T1	c-15t
G1-57	750	common	S450L	S315T1	-
G1-58	14	rare	S450L	-	ΔWT1
G1-59	444	common	S450L	-	c-15t
G1-60	154	less common	S450L	-	-
G1-61	4	rare	S450L	-	c-15t*
G1-62	7	rare	S450L	S315T1*	-
G1-63	1	orphan	S450L	S315T1*	t-8a*
G1-64	3	rare	S450L	S315T1*	c-15t*
G1-65	3	rare	-	Δ	-
G1-66	6	rare	-	ΔWT	-
G1-67	5	rare	-	S315T2	-
G1-68	95	less common	-	S315T1	c-15t
G1-69	1	orphan	-	S315T1	t-8c
G1-70	532	common	-	S315T1	-
G1-71	1	orphan	-	S315T1	c-15t*
G1-72	214	common	-	-	ΔWT1
G1-73	474	common	-	-	c-15t
G1-74	1	orphan	-	-	ΔWT2
G1-75	8	rare	-	-	t-8a
G1-76	11	rare	-	-	t-8c
G1-77	2	rare	-	-	t-8c*
G1-78	9	rare	-	-	c-15t*
G1-79	21	rare	-	S315T1*	-
G1-80	4	rare	-	S315T1*	c-15t*
G1-81	1	orphan	S450L*	S315T1	-
G1-82	1	orphan	S450L*	-	c-15t
G1-83	3	rare	S450L*	-	-
G1-84	4	rare	S450L*	-	c-15t*

G1-85	5	rare	S450L*	S315T1*	-
G1-86	2	rare	S450L*	S315T1*	c-15t*
G1-87	2	rare	H445D*	S315T1	-
G1-88	3	rare	H445D*	-	-
G1-89	2	rare	H445Y*	-	-
G1-90	1	orphan	D435V*	S315T1	-
G1-91	2	rare	D435V*	-	-
G1-92	8	rare	D435V*	S315T1*	-
G1-93	1	orphan	D435V*	S315T1*	c-15t*
G1-94	1	orphan	D435V*; S450L*	S315T1	-
G1-95	1	orphan	D435V*; S450L*	S315T1*	-
G1-96	1	orphan	D435V*; H445D*	S315T1*	-

* Heteroresistant cases: mutation probe as well as the corresponding *wild type* probe stain positive on the strip. G1, first-line genotype; ΔWT, absence of *wild type* band; Δ, complete absence all bands of the *katG* locus, including the Locus Control band; -, absence of mutation; No., number of isolates with the respective genotype.
