# Supplementary material for: Genetic surveillance of first- and second-line drug-resistant isolates of Mycobacterium tuberculosis in Peru
Source: PLoS One. 2026 Jul 9;21(7):e0352881. doi: 10.1371/journal.pone.0352881 (PMC13349105; doi:10.1371/journal.pone.0352881)
Supplement: S5 Table — (PDF) [file pone.0352881.s006.pdf]

S5 Table. Complete set of second-line resistant Genotypes obtained through GenoType MTBDR*sl* v2.

Genotype Code	No.	Category	Mutation			
			gyrA	*gyrB*	*rrs*	*eis*
G2-1	6	rare	ΔWT1	-	a1401g	-
G2-2	22	less common	ΔWT1	-	-	-
G2-3	1	orphan	ΔWT1; D94G*	-	-	-
G2-4	2	rare	A90V; D94G	-	-	-
G2-5	1	orphan	ΔWT2	-	-	-
G2-6	1	orphan	ΔWT2	-	a1401g*	c-14t*
G2-7	3	rare	S91P	-	a1401g	-
G2-8	1	orphan	S91P	-	ΔWT2	-
G2-9	32	less common	S91P	-	-	-
G2-10	4	rare	S91P	-	a1401g*	-
G2-11	1	orphan	A90V	N538D	-	-
G2-12	4	rare	A90V	-	ΔWT1	-
G2-13	42	less common	A90V	-	a1401g	-
G2-14	6	rare	A90V	-	-	ΔWT1
G2-15	1	orphan	A90V	-	-	c-14t
G2-16	150	common	A90V	-	-	-
G2-17	1	orphan	A90V	-	-	c-14t*
G2-18	3	rare	A90V	-	a1401g*	-
G2-19	2	rare	A90V	-	a1401g*	c-14t*
G2-20	1	orphan	ΔWT3	ΔWT	a1401g	-
G2-21	12	rare	ΔWT3	-	a1401g	-
G2-22	2	rare	ΔWT3	-	-	c-14t
G2-23	9	rare	ΔWT3	-	-	-
G2-24	2	rare	D94H	-	ΔWT1	-
G2-25	1	orphan	D94H	-	-	c-14t
G2-26	5	rare	D94H	-	-	-
G2-27	1	orphan	D94H	-	a1401g*	-
G2-28	1	orphan	D94G	ΔWT	a1401g	-
G2-29	1	orphan	D94G	-	ΔWT1	-
G2-30	64	common	D94G	-	a1401g	-
G2-31	1	orphan	D94G	-	ΔWT2	-
G2-32	5	rare	D94G	-	g1484t	-
G2-33	1	orphan	D94G	-	-	ΔWT2
G2-34	1	orphan	D94G	-	-	c-14t
G2-35	131	common	D94G	-	-	-
G2-36	1	orphan	D94G	-	-	c-14t*
G2-37	1	orphan	D94G	-	a1401g*	-
G2-38	1	orphan	D94G; D94H	-	a1401g	-

G2-39	1	orphan	D94N/Y	-	ΔWT1	-
G2-40	1	orphan	D94N/Y	-	a1401g	ΔWT3
G2-41	81	common	D94N/Y	-	a1401g	-
G2-42	1	orphan	D94N/Y	-	g1484t	-
G2-43	66	common	D94N/Y	-	-	-
G2-44	1	orphan	D94N/Y; D94H	-	a1401g	-
G2-45	2	rare	D94N/Y; D94H	-	-	-
G2-46	5	rare	D94N/Y; D94G	-	-	-
G2-47	1	orphan	D94N/Y; D94G	-	a1401g*	-
G2-48	5	rare	D94A	ΔWT	a1401g	-
G2-49	1	orphan	D94A	ΔWT	-	-
G2-50	14	less common	D94A	-	a1401g	-
G2-51	6	rare	D94A	-	-	ΔWT2
G2-52	105	common	D94A	-	-	-
G2-53	2	rare	D94A	-	a1401g*	-
G2-54	1	orphan	D94A; D94G	-	-	-
G2-55	1	orphan	D94A; D94G	-	a1401g*	-
G2-56	1	orphan	A90V; D94H	-	a1401g	-
G2-57	4	rare	A90V; D94G	-	-	-
G2-58	1	orphan	A90V; D94A	-	-	-
G2-59	22	less common	-	ΔWT	a1401g	-
G2-60	35	less common	-	ΔWT	-	-
G2-61	1	orphan	-	ΔWT	-	c-14t*
G2-62	1	orphan	-	E540V	-	-
G2-63	2	rare	-	N538D	a1401g	-
G2-64	13	less common	-	N538D	-	-
G2-65	20	less common	-	-	ΔWT1	-
G2-66	217	common	-	-	a1401g	-
G2-67	13	less common	-	-	-	c-14t
G2-68	3	rare	-	-	-	c-14t*
G2-69	3	rare	-	-	g1484t*	-
G2-70	18	less common	-	-	a1401g*	-
G2-71	3	rare	-	-	a1401g*	c-14t*
G2-72	2	rare	-	E540V*	-	-
G2-73	1	orphan	-	N538D*	a1401g	-
G2-74	3	rare	-	N538D*	-	-
G2-75	2	rare	D94H*	-	-	-
G2-76	1	orphan	D94G*	ΔWT	a1401g	-
G2-77	26	less common	D94G*	-	-	-
G2-78	2	rare	D94G*	-	a1401g*	-
G2-79	6	rare	D94N/Y*	-	-	-
G2-80	1	orphan	D94N/Y*	-	a1401g*	-
G2-81	1	orphan	D94N/Y*	N538D*	a1401g	-
G2-82	1	orphan	D94N/Y*; D94H*	-	-	-
G2-83	1	orphan	D94N/Y*; D94G*	-	-	-
G2-84	6	rare	D94A*	-	-	-

G2-85	6	rare	S91P*	-	-	-
G2-86	1	orphan	S91P*	-	a1401g*	-
G2-87	3	rare	S91P*; D94G*	-	-	-
G2-88	1	orphan	S91P*; D94G*	N538D*	-	-
G2-89	1	orphan	S91P*; D94N/Y*	-	-	-
G2-90	3	rare	A90V*	-	a1401g	-
G2-91	21	less common	A90V*	-	-	-
G2-92	1	orphan	A90V*	-	a1401g*	-
G2-93	1	orphan	A90V*	E540V*	-	-
G2-94	1	orphan	A90V*	N538D*	-	-
G2-95	1	orphan	A90V*; D94G*	-	a1401g	-
G2-96	2	rare	A90V*; D94G*	-	-	-
G2-97	1	orphan	A90V*; D94H*	-	-	-
G2-98	1	orphan	A90V*; D94N/Y*	-	-	-
G2-99	1	orphan	A90V*; D94N/Y*; D94G*	-	-	-
G2-100	4	rare	A90V*; D94A*	-	-	-
G2-101	1	orphan	A90V*; S91P*	-	-	-

* Heteroresistant cases: mutation probe as well as the corresponding *wild type* probe stain positive on the strip. G2, second-line genotype; ΔWT, absence of *wild type* band; -, absence of mutation; No., number of isolates with the respective genotype.
