# Supplementary material for: Genetic surveillance of first- and second-line drug-resistant isolates of Mycobacterium tuberculosis in Peru
Source: PLoS One. 2026 Jul 9;21(7):e0352881. doi: 10.1371/journal.pone.0352881 (PMC13349105; doi:10.1371/journal.pone.0352881)
Supplement: S6 Table — (PDF) [file pone.0352881.s007.pdf]

S6 Table. Temporal variation in the frequency of common drug-resistant genotypes of *Mycobacterium tuberculosis* in Peru, 2019–2022, based on observed and adjusted counts.

Common genotypes	2019		2020		2021		2022		*p*
	O	A	O	A	O	A	O	A	
**G1-57**	229	162	180	173	168	168	173	151	0.64917
**G1-70**	166	117	98	94	121	121	147	128	0.12981
**G1-73**	138	97	85	82	97	97	154	135	0.00185
**G1-59**	128	90	126	121	95	95	95	83	0.03705
**G1-18**	118	83	97	93	69	69	95	83	0.31294
**G1-72**	51	36	47	45	50	50	66	58	0.14527
**G2-66**	77	54	41	39	56	56	43	38	0.11775
**G2-16**	46	32	35	34	37	37	32	28	0.72786
**G2-35**	43	30	32	31	28	28	28	24	0.79697
**G2-52**	34	24	26	25	20	20	25	22	0.88528
**G2-41**	22	16	26	25	14	14	19	17	0.27371
**G2-43**	24	17	14	13	14	14	14	12	0.80125
**G2-30**	34	24	9	9	15	15	6	5	0.00147

O: observed, A: adjusted. *p*: chi-square p-value. G1, first-line genotype. G2, second-line genotype.
